# Recent Advances and Trends of Nanofilled/Nanostructured Epoxies

**DOI:** 10.3390/ma13153415

**Published:** 2020-08-03

**Authors:** Mariaenrica Frigione, Mariateresa Lettieri

**Affiliations:** 1 Department of Innovation Engineering, University of Salento, Prov. le Lecce-Monteroni, 73100 Lecce, Italy; 2 CNR—SPIN, via Giovanni Paolo II 132, 84084 Fisciano (Salerno), Italy; mariateresa.lettieri@cnr.it

**Keywords:** epoxy nanocomposite, dispersion, functionalization, nanofiller, nanoparticles, nanotubes, organic–inorganic hybrid materials

## Abstract

This paper aims at reviewing the works published in the last five years (2016–2020) on polymer nanocomposites based on epoxy resins. The different nanofillers successfully added to epoxies to enhance some of their characteristics, in relation to the nature and the feature of each nanofiller, are illustrated. The organic–inorganic hybrid nanostructured epoxies are also introduced and their strong potential in many applications has been highlighted. The different methods and routes employed for the production of nanofilled/nanostructured epoxies are described. A discussion of the main properties and final performance, which comprise durability, of epoxy nanocomposites, depending on chemical nature, shape, and size of nanoparticles and on their distribution, is presented. It is also shown why an efficient uniform dispersion of the nanofillers in the epoxy matrix, along with strong interfacial interactions with the polymeric network, will guarantee the success of the application for which the nanocomposite is proposed. The mechanisms yielding to the improved properties in comparison to the neat polymer are illustrated. The most important applications in which these new materials can better exploit their uniqueness are finally presented, also evidencing the aspects that limit a wider diffusion.

## 1. Introduction

The epoxy resin family comprises thermosetting polymers characterized by many outstanding characteristics, such as excellent mechanical strength and stiffness, chemical and corrosion resistance, durability, and stability if exposed to different environmental agents, low shrinkage upon curing process, excellent adhesion to different materials [1]. They are extensively applied in many diverse applications and industrial fields as adhesives and coatings, matrices to produce fiber-reinforced composites, electrical components, and insulators [2].

The already good properties of epoxy resins can be further enhanced with the addition of one or more nanosized phases, obtaining the so-called nanocomposite, or with the realization of a system composed by an organic (i.e., epoxy) phase strictly interconnected at the nano-scale level to an inorganic one, the so-called organic–inorganic (O–I) hybrid systems. O–I hybrid materials are, in fact, often referred to as structures characterized by nano-scale repeating distances between the organic phase and the inorganic one [3].

Along with many withstanding properties, epoxy resins also display some weaknesses, such as very low values of toughness (they are brittle in nature, like other thermosetting polymers), low thermal/electrical conductivity, high coefficients of thermal expansion, and deficiencies that somehow restrict their use in some more demanding applications (nano-electronic, medical devices, aeronautical applications). These properties can be on-demand modified, improved, or adjusted to address specific requirements again with the addition of preformed nano-sized constituents, i.e., the nanofillers.

**Figure 1:**
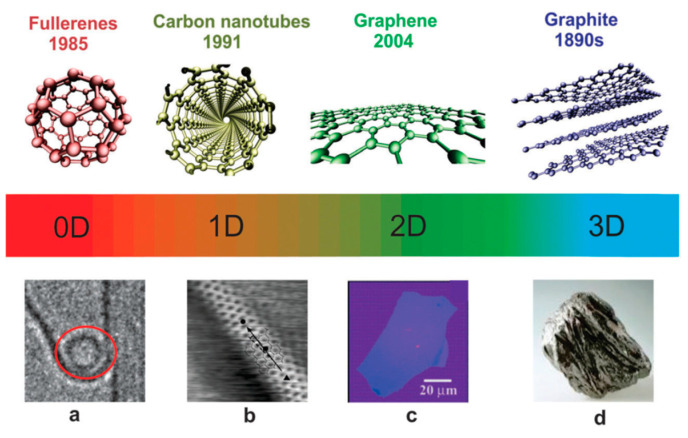
Classification and examples of nanomaterials with 0D, 1D, 2D, and 3D structures: (**a**) fullerene (inside red circle); (**b**) single-walled carbon nanotube; (**c**) multilayer graphene flake, and (**d**) natural graphite. Reprinted with permission [4].

Nanofillers can be defined as materials of different chemical nature that have at least one dimension between 1 and 100 nm, produced as 0D, 1D, 2D, and 3D nanomaterials (Figure 1).

The addition of nanoparticles, possessing different chemical structures, dimensions, and shape, can improve or properly modify specific characteristics of the hosting epoxy matrix; these remarkable improvements arise from the very high surface/volume ratio of the nanomaterials, leading to multipurpose “smart” functional nanocomposite. It must also be bared in mind that the chemical composition of the particle surface, which can be different from the internal one, has a strong effect on the interaction between the nanofiller and the epoxy matrix.

**Figure 2:**
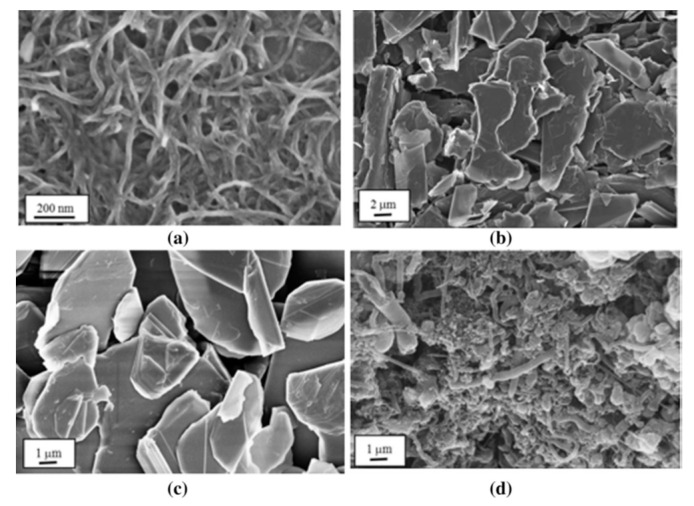
SEM images of nanofillers in an epoxy matrix: (**a**) carbon nanotubes; (**b**) graphene nanoplatelets; (**c**) boron nitride nanosheets; and (**d**) boron nitride nanotubes. Reprinted with permission [6].

In addition to their chemical composition, the size and the aspect ratio of the nanofillers as well as their loading content in the epoxy matrix are all key parameters that severely affect the final performance of the nanocomposite [5]. The nanofiller compound can be made up of nanoparticles (for instance, minerals), nano-sized sheets, or platelets (e.g., boron nitride, graphene oxide) or nanofibers (such as carbon nanotubes or electrospun fibers); Figure 2 shows the morphology of different nanofiller types.

The shape and the features of the nanofiller will also define the process employed for the production of the nanocomposite. The nanophases are typically dispersed in the (un-cured liquid) epoxy matrix in very low percentages (most likely never above 10 wt.%), taking into account that the best performance is always obtained at low loads of the nanofiller. This solution also allows for reduction of both the aggregation effect of the nanoparticles and the final cost of the nanocomposite. 

**Figure 3:**
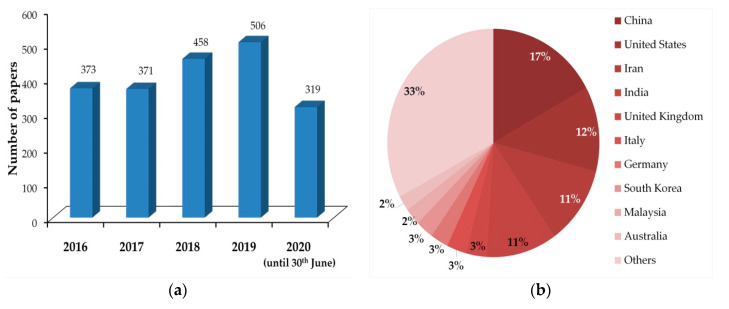
Papers dealing with epoxy nanocomposites, published between 2016 and 30 June 2020, found in the Scopus database: (**a**) papers per year; (**b**) authors’ affiliation per country.

International research is particularly active in this field, as witnessed by a huge number of papers that appeared in the last five years on this challenging topic. We found, in fact, 2027 papers on the Scopus database looking for the keyword “epoxy nanocomposite”. Over time, the number of published papers per year shows an increasing trend (Figure 3a). From the affiliations of the authors, we counted four countries as the most productive (more than 250 published papers): China, USA, Iran, and India (Figure 3b).

A comprehensive review of all the papers published in the last five years on the polymer nanocomposites based on epoxy resins is outside of our purposes. Rather, this manuscript aims at giving an overview on the different trendy nanofillers that have been successfully added to epoxies to enhance some of their characteristics, in relation to the nature and the feature of each nanofiller. Another class of nanostructured epoxies, i.e., the organic–inorganic hybrid one, is introduced, highlighting its strong potential in many applications. Differently from other recent reviews focused on specific properties of the nanocomposites [7,8,9,10] or individual nanoparticles [11,12,13] added to epoxy, a wide-ranging overview of many characteristics and nanofillers has been presented in this paper. The different methods and routes employed to produce nanofilled/nanostructured epoxies are then described. The main applications in which these new materials can better exploit their uniqueness are finally presented, evidencing, at the same time, the aspects limiting their wide commercial exploitation.

## 2. Nanofillers for Epoxy Resins

Different nanoparticles have been recently experimented as fillers able to enhance/modify properties and characteristics of epoxy resins. They belong to different chemical families, i.e., carbon-based fillers, metal oxides, and clay nanoparticles, just to mention a few of them.

### 2.1. Carbon-Based Nanofillers

This family of nanofillers includes several compounds already widely used at very low contents in epoxy-based nanocomposites intended for many different applications, due to a mix of desirable properties, such as excellent physical and mechanical characteristics, hardness, high thermal and electrical conductivity (the latter being a strategic feature, considering that epoxy, as the majority of polymers, is intrinsically an insulator, with an electrical conductivity from 10^−10^ to 10^−14^ S/cm [14]), chemical stability, and biocompatibility (in the case of nanodiamonds) [15].

**Figure 4:**
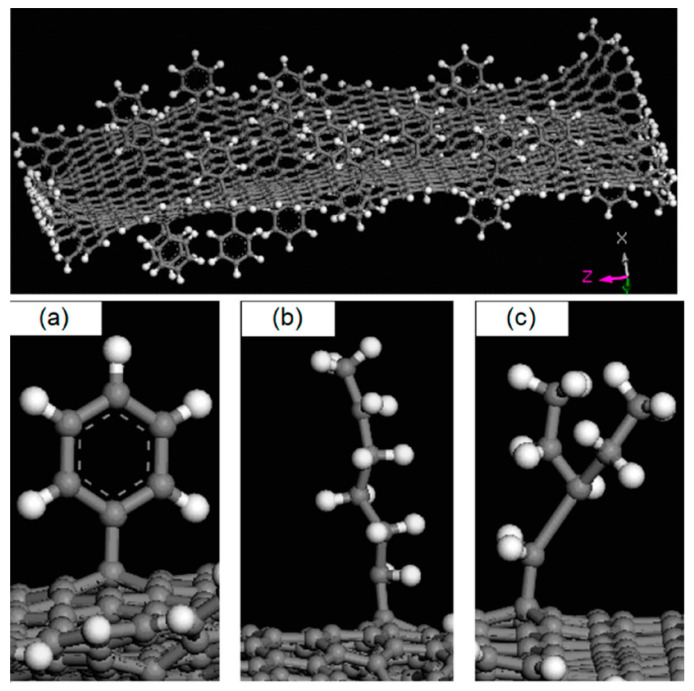
Illustration of graphene with different functionalized groups (**a**) phenyl groups (**b**) –C_6_H_13_ and (**c**) –C_2_H_4_(–C_2_H_5_)_2_. Reprinted with permission [27].

Graphene (Figure 4), with its most important derivative graphene oxide (GO), was probably the first compound of this broad family proposed to manufacture high-performance nanocomposites due to its unique shape (two-dimensional crystals with an average thickness of about 1^−10^ m and diameter 0.5–5 μm) and outstanding electrical (with a conductivity of 6 × 10^5^ S/m), mechanical (in terms of tensile strength and especially Young’s modulus, the latter being equal to 1.1 TPa), thermal (conductivity around 5000 W/m·K), and barrier properties [16]. Its addition can provide a reinforcing effect to the epoxy matrix and multifunctionality for the most varied engineering applications (anticorrosive coatings, structural adhesives, thermal conductors) at lower costs than the more expensive carbon nanotubes, to fabricate electric and electronic devices, and also in the field of fiber-reinforced polymers [17,18,19,20,21,22,23]. GO nanosheets, characterized by an extremely high surface area (~2630 m^2^/g), may represent a feasible alternative to functionalized/unmodified carbon nanotubes also when enhancements in mechanical, electrical, and thermal properties are mostly required for a conventional epoxy [24]. Graphite carbon nitride (g-C_3_N_4_), an advanced derivative of graphene, is able to provide even greater anticorrosion performance to waterborne epoxy due to the strong shielding effect of the surface modified g-C_3_N_4_ nanosheets [25]. In the field of adhesives, functionalized graphene oxides (i.e., based on graphene oxide-ferric and graphene oxide-ferric dihydrogen phosphate) have been employed as hardeners for cold-curing one-component epoxy nanocomposites [26]. A substantial increase (greater than 144%) in lap shear strength over the pristine resin was achieved, along with an additional acceleration effect on the curing reactions, attributed to the chemical nature and the morphology of the functionalized GO.

Carbon nanotubes (CNTs), characterized by low density and high aspect ratio (diameter even less than 1 nm and length of dozens of nanometers), are among the most promising nanoreinforcements for many polymers, including the epoxy resins, due to their capacity to positively modify the mechanical, electrical, rheological (key parameter for the processability), and thermal properties of the hosting polymeric matrix, even at very low percentages (0.1–0.2 wt.%); the possibility to use very low amounts of nanofiller is desirable in order to limit self-aggregation of the nanoparticles [28,29]. On the other hand, chemical functionalization of the nanoparticle surfaces allows adding greater amounts (of one order of magnitude) of CNTs in the epoxy, thus enhancing further the thermal and mechanical properties of the nanocomposite [30,31]. Grafting carbon nanotubes on the surface of carbon fibers enables them to also exploit their remarkable properties in the field of fiber-reinforced polymer (FRP) composites, improving the bond at the fiber/matrix interface and enhancing the resistance to debonding during mechanical stress, even though with this procedure, the wettability of the fibers can be affected by the addition of high amounts of CNTs [32]. A proper functionalization of CNTs can still improve their dispersion in the epoxy matrix of the FRP, leading to a substantial growth of mechanical performance and impact resistance of the composite without impairing the wettability of the fibers [33].

A variant of CNTs is represented by multi-walled carbon nanotubes (MWCNTs), possessing an average outer diameter from 5 to 50 nanometers and a length of 0.5–200 µm, that demonstrated to give the epoxy resin, and the relative FRPs, excellent mechanical, thermal, and durability characteristics [34,35,36,37]. It is also worth mentioning that the addition of montmorillonite or silica nanoparticles to MWCNT aimed to obtain epoxy-based hybrid composites with exceptional properties, due to the synergistic effect raised from both the combination of different nanofillers and the interactions between them [38,39]. Similarly, epoxy-based nanocomposites containing MWCNTs decorated with MnFe_2_O_4_ nanoparticles (average diameter around 30 nm) are very appealing for electrical/electromagnetic applications, also due to the high percolation threshold that can be achieved in MnFe_2_O_4_ [40]. Finally, the fire resistance of epoxy nanocomposite can be enhanced upon the addition of medium amounts (i.e., 15 wt.%) of MWCNTs functionalized through the grafting of phosphate melamine salt on their surface [41].

Nanodiamond is another nanomaterial incorporated into epoxy resins to enhance the mechanical and tribological properties of the composite [42]. Functionalization is often necessary to obtain good performances [43,44]. Nanodiamonds (NDs, with particle size in the range 4–10 nm) functionalized with ozone/tetra-ethylene pentamine has been successfully employed as a reinforcing agent (at an NDs optimal content of 0.50 wt.%) to produce epoxy nanocomposites with improved fracture resistance and thermal conductivity [45].

MXenes is a family of 2D transition metal carbides, carbonitrides, and nitrides. Due to their outstanding mechanical, thermal, and electrical properties, these multilayered compounds have been employed to realize multifunctional epoxy-based nanocomposites characterized by enhanced electrical and thermal conductivity, self-healing characteristics, excellent toughness, and mechanical properties, even at low filler percentages (1 wt.%) [46,47,48,49,50].

### 2.2. Metal Oxides and Other Nanooxides: Al_2_O_3_, TiO_2_, SiO_2_, ZnO, Fe_3_O_4_ Nanoparticles

**Figure 5:**
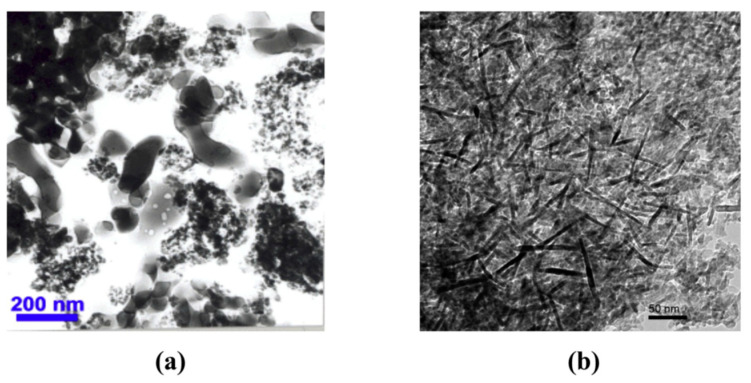
TEM images of nanoalumina as (**a**) nanospheres and (**b**) nanorods. Reprinted with permission [51].

Among the other metal oxides, nano-Al_2_O_3_ is certainly one of the most widely used to manufacture polymer nanocomposites with enhanced thermal and mechanical performance. A further favorable advantage resides in the lower production costs of alumina nanoparticles if compared to carbon-based nanofillers and nanotitania. The average size (diameter) of this nanofiller, which can be found as nanospheres or nanorods (Figure 5), is in the range of 10–80 nm.

The positive effects of the nano-Al_2_O_3_, especially on mechanical properties (the nanoparticles are able to significantly increase the strength at the fiber/matrix interface [52]), can be found also when these particles are added in fiber reinforced composites based on an epoxy matrix, even at very low contents (i.e., 1–0.5 wt.%) [53,54,55]. The beneficial effects of nanoalumina are also reported in the field of epoxy adhesives [51,56,57]. As in any other application of nanocomposites, a key role is played by the effective dispersion of nanoparticles inside the epoxy matrix. The inclusion of Al_2_O_3_ nanoparticles is also able to improve the durability of the composites against harsh environmental agents [54].

Due to their strong adhesion exerted to a wide range of surfaces and their good chemical resistance, epoxy resins are frequently used as anticorrosive and protective coatings for metals. The addition of a nanofiller in an epoxy-based coating can be a good choice to enhance the scratch resistance of the coating, thus improving the durability and the performance of the coating. On the other hand, the special shape/size of the nanofiller can be used to carry corrosion inhibitors, released on the basis of the surrounding pH value, the direct addition of which in the epoxy resin can lead to a decrease in their efficiency [58]. This is the case of titania nanotubes.

Titania is the common name of titanium dioxide (TiO_2_), a nanofiller for coatings that is especially popular for its high corrosion resistance, chemical stability, and non-toxicity coupled with affordable costs. The addition to epoxy of titanium dioxide nanotubes, i.e., nanocarriers encapsulating an organic inhibitor for corrosion, is able to impart improved resistance to corrosion to the final coatings [59]. The formation of a nanotitania phase in an epoxy hybrid network, accomplished with a sol-gel method, is able to enhance the performance of epoxy-based coatings designed to also protect different substrates, such as the stone of monuments and ancient constructions [60,61]. The effectiveness of nano-TiO_2_ in improving the durability of an epoxy matrix is also confirmed in other studies concerning composites based on glass fibers (GFRP); the addition of 0.1 wt.% of this nanofiller is able to reduce the coefficient of water diffusion by 9%, with additional improvements in flexural and interlaminar strengths [62].

SiO_2_ are well-known nanoparticles (with average diameter of 10–30 nm) able to improve the most important functional properties of the coatings for metals, such as abrasion and scratch resistance, especially when their surfaces are properly modified [63]. The inclusion of such nanoparticles in epoxy-based coating formulations is capable to improve to a large extent the corrosion resistance of metallic (steel) surfaces, especially at a medium (i.e., 5 wt.%) filler content [64]. The applications of epoxy resins as adhesives take advantage with the addition of a similar amount of nanosilica (i.e., 6 wt.%), also when analyzing the response to dynamic/cyclic loads with fatigue tests [56]. Referring to the structural applications involving FRP, effective interfacial interactions of silica nanoparticles with the epoxy matrix of a reinforced composite can also lead to significant enhancements of its mechanical (tensile and flexural strength), vibration, and damping characteristics [65]. Furthermore, the addition of even low loads (1–2.5 wt.%) of epoxy-grafted nanosilica brings about enhancements in mechanical and dielectric strength, as well as reductions in the water uptake [66].

Nanostructured organic–inorganic (O–I) “hybrid” systems containing nanosilica can be formed directly in the epoxy network during its development, with a morphology consisting of co-continuous organic domains chemically linked to the inorganic phase. These systems gained a large success due to their outstanding synergistic properties derived from the beneficial combination of nanosized phases into a bulk epoxy, differing in chemical composition, physical properties, and morphology of the final phases. They can find an advantageous application, for instance, in the field of “cold-cured” adhesives, i.e., those able to set and harden at ambient temperature, due to a marked improvement in properties, in particular the glass transition temperature and durability [67,68].

ZnO-based nanofillers are used for reinforcement of epoxy matrices; increased surface hardness [69] and enhanced corrosion resistance [70] have been found in such nanocomposites.

Finally, Fe_3_O_4_ nanoparticles (diameter ranging from 10 to 50 nm) have been successfully experimented with to produce magnetic epoxy-based nanocomposites for a wide range of applications, such as microwave absorbing elements, microelectromechanical devices, micro-sensors, and systems to store/harvest energy [71,72]. A distinctive advantage of such nanomaterials in the mentioned applications resides in the possibility to control the final properties by adjusting the nanofiller content in the epoxy matrix. It has been reported, for instance, that the nano-Fe_3_O_4_ content has a positive effect on the capacity to store energy, on magnetic properties, and on microwave absorption performance [71].

### 2.3. Nanoclays

Originally employed mainly in thermoplastic nanocomposites, nanoclays are now also widely employed in epoxy systems with different functions, i.e., as a thermal stabilizer, to supply electrical and optical properties, flame retardancy, biocompatibility, and as scavengers for pollutants. Nanoclays, in addition, are able to confer enhanced mechanical properties, corrosion resistance [73], and fire resistance to the epoxy matrix of FRP composites based on glass fibers, even at low percentages (1 wt.%) [74,75].

Halloysite (an aluminosilicate clay, Al_2_Si_2_O_5_(OH)_4_) nanotubes (HNTs), display inner/outer diameters around 12–15/50–60 nm and a length in the range 0.5–10 μm. HNTs are characterized by outstanding biocompatibility, thermal stability, and mechanical strength, and constitute promising nanofillers for the development of epoxy-based nanocomposite for many different applications [76]. Their unique nature depends on the different natures of inner (largely composed by SiO_2_) and outer (mainly Al_2_O_3_) surfaces of the nanotubes. Low contents of halloysite nanotubes (up to 1 wt.%) are preferred, leading to increased cross-linking density in comparison to the pristine epoxy, while at higher loads (i.e., 2 wt.% or greater), the advancement of curing reactions is somehow hindered.

### 2.4. Phosphorus-Based Nanofillers

The addition of nanofillers based on phosphorus compounds to epoxy systems has been studied as a means to enhance their resistance to fire. The advantages of such halogen-free nanomaterials reside in their low toxicity, in the formation of a stable carbonaceous (char) layer during a fire, limiting further advancement of flame, with low emissions of smoke [77]. 

Aluminum hypophosphite nanoparticles (possessing a diameter lower than 60 nm) are phosphorus-based compounds that can be used as a flame retardant additive for epoxy resins, even at very low percentages (up to 0.5 wt.% of epoxy) and without any superficial modification [78]. Phosphorene, a 2D material used as nanofiller, also exhibits high flame retardant efficiency, even when small amounts are added [79,80].

Similarly, the addition of nanoaluminum diethylphosphinate to low amounts of functionalized MWCNTs is able to enhance the fire resistance of epoxy resin nanocomposites [81].

### 2.5. Other Nanoparticles

Several recent studies in the literature describe the addition of boehmite, i.e., colloidal plate-like crystalline nanoparticles (with average thickness around 15 nm), in epoxy resins. Boehmite (γ−AlO(OH) is a mineral aluminum hydroxide in which the Al cations are placed between octahedral oxygen layers. It has been demonstrated that this stiff nanofiller supplies a noticeable reinforcing effect to the host epoxy matrix, with enhancements in elastic modulus and strength as well as in fracture toughness and hardness [82,83,84]. In addition, bohemite nanofillers are able to enhance, to a large extent, those mechanical properties dominated by the polymeric matrix of fiber-reinforced polymers [84]. The observed positive features are likely to derive from a combination of chemical and physical interactions taking place at the interface between the epoxy matrix and the nanoparticles, the latter enhanced by chemical modifications (i.e., functionalization) of the nanofiller surface. The amount of the added nanofiller also plays a relevant role in modifying the properties and the curing kinetics of the nanocomposite. As an example, even though appreciable enhancements in stiffness are obtained at not-low boehmite nanoparticles loads (i.e., up to 15 wt.%), these amounts do not allow to achieve an acceptable cross-linking density [85]. At lower amounts (3 wt.%) of boehmite, on the other hand, increases in mechanical (maximum strength and Young modulus in flexural mode) and surface (hardness) properties are observed (up to 35%), along with an appreciable increase in *T*_g_ [83].

Boron nitride (BN) can be added in forms of nanosheets (65–75 nm thick) in epoxy matrices to impart high thermal conductivity to these polymers, due to the high thermal conductivity (around 300 W/m·K) of BN in isolation [5]. BN also presents other good characteristics, such as a low thermal expansion coefficient, a stable crystalline structure, a low dielectric constant, and nontoxicity, which make this nanofiller particularly suitable in combination with the epoxy. On the other side, a very high content of this filler (>60 vol.%) is required to achieve an adequate thermal conductivity value in the cured epoxy; a very high loading of the nanofiller, in addition, produces a substantial increase in the viscosity of the uncured resin-filler mixture, which limits the occurrence of a homogeneous dispersion of nano-BN in the epoxy matrix. To overcome these problems, Al_2_O_3_ nanoparticles can be incorporated in the epoxy matrix along with BN platelets; the formation of a sort of bridge between the different nanofillers is able to enhance the thermal conductivity of the nanocomposite, creating a conductive pathway [5]. The total percentage of the nanofiller materials in the composite never exceeded 30 wt.%; it was, therefore, possible to achieve the improved thermal properties without impairing the viscosity of the uncured resin—that is to say, its processability. In the field of electronic applications, the incorporation in an epoxy matrix of BN nanosheets (BNNSs) superficially modified with silver nanoparticles (AgNPs) allows obtaining a binary nanofilled composite (at 25 vol.%) possessing contemporarily high thermal conductivity and electrical insulation values, exploiting the complementary features of BNNSs and AgNPs [86]. With a properly designed approach to achieve the modification of BNNSs, the electrical insulation properties of epoxy nanocomposites are not reduced by the addition of the nanofiller with high conductivity, i.e., silver nanoparticles, since these are effectively blocked on the surface of electrically insulated BN nanosheets. Similar features can also be obtained by combining multi-layer graphene and BNNSs, using silver nanoparticles as a bridging agent, obtaining an excellent thermally stable epoxy nanocomposite [87]. Finally, a multifunctional nanofiller, based on boron nitride nanosheets functionalized with ionic liquid, has been proposed as an additive which acts simultaneously as a curing agent and flame retardant for the epoxy matrix [88].

Aluminum nitride (AlN) spherical nanoparticles (average diameter of 50 nm) were added to epoxy resin in the view to enhance its electrical properties; these nanocomposites display, additionally, a greater thermal resistance [89]. 

Among nanofillers experimented for the fabrication of piezoelectric nanosensors and energy harvesting devices, produced in the form of epoxy-based FRP elements, antimony sulfoiodide (SbSI) nanowires are worthy to be mentioned due to their high value of electromechanical and piezoelectric coefficients [90].

## 3. Production of Nanocomposites

Polymer-based nanocomposites differ from conventional (macro/micro) composite macromolecular materials, especially for the substantial larger interfacial area between the matrix and the reinforcing nanophase(s) (typically an order of magnitude greater than for conventional composites) and for the outstanding aspect ratio of the nanofiller. A high outer surface of the nanoreinforcement implies that the addition of a relatively small amount of this filler in the polymeric (epoxy) matrix enables appreciable modifications in the macroscopic properties of the nanostructured material.

**Figure 6:**
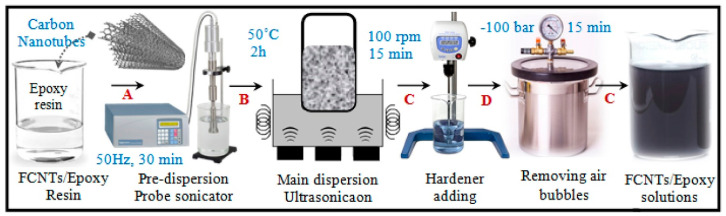
Example of preparation of a functionalized carbon nanotube/epoxy solution. Reprinted with permission [33].

The success of the nanocomposite, in terms of achievement of optimized properties, on the other hand, relies on an effective dispersion of the nanophase(s) in the epoxy matrix [7]. To this aim, the nanofiller must be added into the liquid resin (usually indicated as “Part A”) prior to curing, and very efficiently mixed in the matrix to achieve a homogeneous system, in which the nanofiller is uniformly and finely dispersed in the resin. This task can be facilitated by the use of mechanical methods (e.g., magnetic stirring, ultra-sonication), which are cost-efficient, eco-friendly, and single-step processes. On the other hand, often nanofillers functionalized by physical methods just avoid self-aggregation temporarily. A chemical approach can also be preferred when the mechanical action can alter the structure of the nanomaterials, diminishing their performance [7]. Afterward, the final mixture is cured employing an appropriate curing cycle (of temperature and time) in the desired shape and dimensions. An example of preparation of a nanofilled epoxy solution is schematized in Figure 6. 

The enhancement in the final properties of epoxy nanocomposites is, then, basically governed by two factors, namely the dispersion of the nanofiller and its interaction with the matrix [91]. Therefore, the potential of a nanoreinforcement in a nanocomposite can be not completely exploited due to the possible aggregation of the nanoparticles and/or the development of a scarce interfacial interaction with the epoxy matrix. An effective stress transfer between the epoxy matrix and the embedded nanofiller is governed by their interfacial interactions [28]. The latter include secondary weak bonds, such as van der Waals forces, and the mechanical interlocking due to an uneven surface structure. These weak interfacial interactions, therefore, cannot be able to guarantee an effective stress transfer. Furthermore, the scarce dispersion achieved during the production of the nanocomposite can also lead to ineffective reinforcement. As an emblematic example, the use of graphene as a nanofiller for epoxy nanocomposites is mainly limited by a scarce surface interaction: the strong van der Waals forces among the graphene nanosheets, characterized by a very large surface area, favor their agglomeration in the host matrix. Furthermore, a weak interaction of the epoxy matrix with the nanoparticles can also lead to a little increase in its glass transition temperature. Chemical modifications performed on the nanofiller surface can be a feasible route to improve the dispersion of the nanoparticles in the polymeric matrix, also enhancing the interfacial adhesion with the epoxy phase and, in turn, improving the mechanical properties of the epoxy-based nanocomposite to a greater extent. That is to say: any interaction at the nano-scale level can greatly affect the nanocomposite properties at macroscale level. The grafting of different functional groups on the outer surface of nanofillers is also a route to diversify their purposes. Surface treatments can be performed at the surface of any kind of nanofiller by a proper selection of chemicals able to create organic links, i.e., with a bridging action, between the epoxy and the surface of the nano-sized filler, namely through oxidation, amidation, thiolation, and silation.

Often, the functionalization of the surface of a nanofiller is achieved by treatment with silane solutions, such as in the case of nanoclays [75], graphene [92,93], boron nitride [87], and SiO_2_ nanoparticles [64]. The silane can also act as a coupling agent, allowing the graft of other chains/compounds on the surface of the nanofiller, synergistically improving the nanocomposite performance [22,94].

In the case of CNT, MWCNT, graphene oxide nanoparticles, and surface functionalization can also be obtained through a covalent attachment of amine functional groups on the nanofiller surfaces [24,28,95]. The functional groups were found to contribute also with an additional mechanical interlocking during a deformation. Other possible functional modifications on CNTs and graphene oxide surfaces include grafting of alkyl chains [96], polystyrene sulfonate, aminostyrene [97], zinc oxide [98,99], thiols [21,30], and thiol-ended hyperbranched polymers [31]. Alternative chemical methods that involve the use of non-toxic solvents or a bio-approach have also been proposed [100,101,102,103,104,105]. The modification of the MXenes surface can be achieved by the creation of thermally stable oxygen functional groups, able to assure interfacial adhesion with the epoxy matrix [50]. 

Plasma treatment has been successfully employed to functionalize the surface of carbon-based nanofillers, such as CNTs, GNP (graphene nanoplatelets) [6,106], and of silica nanoparticles [107].

Among the others, surface functionalization treatment employing ozone can be performed under an air atmosphere to provide oxygen radicals on the surface of nanofillers; this can be achieved at reasonable costs and by employing simple equipment [23]. As an example, the functionalization of nanodiamonds through a coupled treatment with ozone and tetra-ethylene pentamine (TEPA) was found to be effective to improve the interfacial interactions with epoxy matrix, enabling achievement of enhanced mechanical and thermal properties [45]. This approach was, in fact, able to avoid any aggregation process of the NDs, thereby exploiting the best interface synergism developed between the nanofiller and the epoxy matrix. 

In some cases, the chemical compounds employed to functionalize the surface of the nanofiller can additionally act as curing and/or toughening agents for the epoxy matrix, thus modifying the kinetics as well as further improving some characteristics of the nanocomposite, in particular the energy absorption, which is a highly desirable feature in some applications, for instance, in the field of adhesives [45].

The organic–inorganic nanostructured epoxy-silica hybrids are produced employing the hydrolysis–condensation technology typical of ceramic materials. In these nanosystems, the inorganic phase is composed of siloxane nanodomains, i.e., nanometric silica with hydroxyl groups produced by the sol-gel method concurrently to the cross-linking reactions of the organic (epoxy) phase [68]. It is then possible to obtain the inorganic nanophase and organic network interconnected at a nano-scale level, able to achieve exceptional performance and capabilities, by appropriately controlling the kinetics of the respective reactions. This approach can also represent a successful solution to the lack of compatibilities and poor interface interactions of the organic–inorganic phases, from which most nanocomposites suffer.

## 4. Curing/Cross-Linking Process

The design stage of epoxy-based nanocomposites able to meet specific requirements includes the selection of a proper nanofiller or an appropriate organic–inorganic hybrid nanostructure and the identification of suitable curing conditions, in order to achieve a microstructure of the nanocomposite able to supply the desired characteristics/properties. On one hand, the key parameter governing an effective adaptation of the nanofilled epoxy microstructure to the intended application is its cross-linking density developed during curing according to appropriate reaction kinetics. Depending on the nanofiller chemical nature and physical characteristics, its addition in the epoxy can sterically hinder curing reactions, thus leading to a decrease in cross-linking density and in *T*_g_, or even enhance both of them. According to their physical and chemical properties, in fact, the nanoparticles can influence the chemistry and the rate of the curing reactions and, in turn, the resulting cross-linking density and glass transition range. It is fundamental, therefore, to monitor the advancement of cure in relation to the selected process parameters able to guarantee the formation of an optimum nanostructure within the epoxy-based nanocomposite.

The addition to a conventional (cured at a moderate temperature) DGEBA (diglycidyl ether of bisphenol A) resin of boehmite nanoparticles (3 wt.%) with organically modified surfaces has brought increased kinetics of cross-linking reactions with appreciable growths in *T*_g_ (about 20 °C) [83]. An increase of cross-linking density in the epoxy network, confirmed by an increase in the glass transition temperature, has been observed for nanocomposites containing aluminum hypophosphite nanoparticles (increase in *T*_g_ greater than 8%) [78]. Increases in *T*_g_ have also been reported for epoxy-based nanocomposites containing epoxy-grafted silica (up to 5–6% at a filler content of 2.5 wt.%) [66] and nano-aluminum nitride (around 13% by adding 1 wt.% of nano-AlN) [89]. On the other hand, decreases in *T*_g_ have been found upon the incorporation of nano-Al_2_O_3_ particles (greater than 10 °C) at a 0.1 wt.% loading [54]; more limited reductions in *T*_g_ (up to 3 °C) have been measured upon addition of boehmite nanoparticles at low concentration levels (i.e., 1 wt.%) [85]. The different effects of the addition of nanofillers on the *T*_g_ of the epoxy matrix are the result of different events, namely the restrictions in epoxy chain movements, due to the presence of nanoparticles, and the change in cross-linking density of the matrix resin. As already underlined, the functional groups employed to modify the surface of the nanofiller, with the aim of avoiding the agglomeration of the particles and improving their dispersion in the epoxy matrix, may also affect (i.e., increase or even decrease) the cross-linking process of the resin and, in turn, the glass transition temperature of the nanocomposite [24]. As an example, the surface chemistry of halloysite nanotubes, properly modified by different functionalization routes, significantly affects the development of the cross-linked network of HNTs-filled epoxy and, in turn, its final properties [76].

## 5. Properties of the Epoxy Nanocomposites

The incorporation of nanoparticles in epoxy resins allows modification of many properties. In comparison to the neat polymer, enhanced mechanical properties [6,16,27,32,35,45,57,69,85,101,106,108,109,110,111,112,113,114,115,116,117,118,119], improved corrosion resistance [21,25,59,64,70,92,108,120,121,122,123,124,125,126], increased thermal and electrical conductivity [5,15,16,17,27,45,86,87,101,109,110,117,127,128], high dielectric permittivity and low dielectric loss [93,107,129,130], and reduced flammability [79,123] are observed.

Shape, size, and dispersion of the nanoparticles, more than the type of nanofiller, influence the final properties of the composite. In fact, nanoparticle aggregation is able to create defects into the matrix which may lead to micro-cracks [27,108,127,128,131]. It has been found that linearly shaped nanoparticles (e.g., CNTs) mainly produce increases in mechanical properties [108] and 2D nanosheets (such as GNP, graphene oxide, and MoS_2_) appreciably improve the barrier properties [92,121,124,125], especially corrosion resistance, while spherically shaped nanofillers (e.g., fullerene), having a low capability to aggregate, yield an overall improvement in nanocomposites’ properties [108,132]. Synergistic effects from using combinations of different nanofillers have been observed [86,106,117], even if the benefits are sometimes negligible [6].

The flexural and tensile strengths of the epoxy nanocomposites are higher (even above 20%) than those measured in the pure resin [39,129]. In most cases, the presence of nanofillers produces increases in toughness [19,106,133], which are larger at high nanoparticles loadings [110]. Stiffness is also enhanced [85,114], but sometimes low increments are observed [110]. Great adhesive strength is due to mechanisms of mechanical nanoanchoring [56,92,110,134]. Furthermore, the addition of nanofillers can improve the resistance to abrasion [63,108] and indentation [69] of the epoxy nanocomposite.

**Figure 7:**
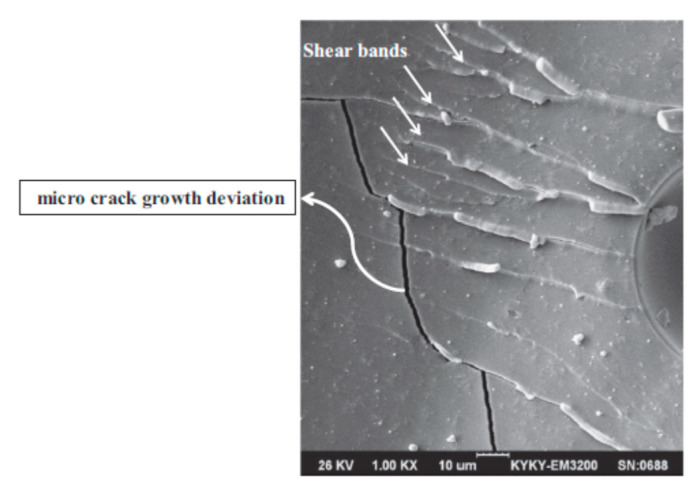
Micro-crack growth deviation in epoxy adhesive reinforced with MWCNTs. Reprinted with permission [38].

Epoxy resins may potentially contain micro-pores generated during the manufacturing process. A certain reduction of voids and pores can be obtained by nanofiller loading. The added nanoparticles can also restrict the movement of polymer molecules under mechanical loads. Both these phenomena result in increases in mechanical properties [69]. Where a crack encounters the nanofillers, propagation can just proceed with higher tilt or twist at greater angles (Figure 7); otherwise, it is locally interrupted [16,38,113].

**Figure 8:**
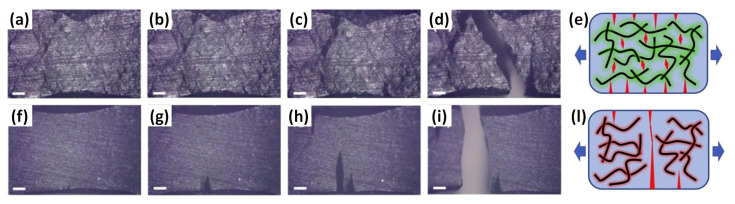
Images of tensile testing on uniformly distributed CNTs in epoxy (**a**–**d**) and agglomerated CNTs in epoxy (**f**–**i**) under different moments (scale bar is 200 mm); (**e**,**l**) possible crack propagation mechanisms. Adapted with permission [101].

A large amount of energy, therefore, is required to allow the crack to pass the uniformly dispersed nanofillers and to propagate until the fracture failure [45,101,113]. This mechanism leads to significant increases in the mechanical properties of the material. Suitable dispersion of the nanofiller is a key factor to obtain nanocomposites with high mechanical properties [17,116]. In those regions where agglomeration of nanoparticles takes place, cracks finds weaker pathways, energetically favorable, and fractures more easily appear [101] (Figure 8).

In addition, when nanofiller agglomeration occurs (especially at high nanofiller contents), the interchain interactions during the cure of the polymer may be hindered, yielding reductions in the tensile and flexural strengths [112,129]. 

It is worth mentioning that the mechanical properties of nanofilled epoxy resins significantly decline as a consequence of water absorption, even at low amounts of absorbed water. This behavior has been ascribed to the weakening of the interface between the epoxy matrix and the nanofillers which hinders the transfer of the stresses under mechanical loads [36,110].

**Figure 9:**
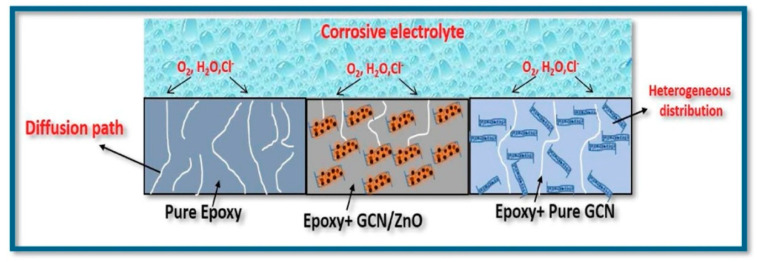
Schematic representation of penetration of aggressive species from a corrosive electrolyte (3.5% NaCl solution) into epoxy coatings with and without GCN/ZnO nanoparticles (GCN: graphitic carbon nitride). Reprinted with permission [70].

Neat epoxy resins usually exhibit low corrosion protection because of their proneness to defect formation and shrinkage, as well as because of their poor flexibility. Well-dispersed nanofillers are able to promote barrier and anti-corrosion performance in epoxy coatings [120,121]. Corrosive media, such as air, moisture, and ions, can penetrate the epoxy coatings, reaching and affecting the metallic substrate. The addition of nanofillers (especially graphene oxide) improves the barrier properties yielding an effective increase in corrosion resistance (Figure 9). In fact, in the nanofilled epoxy coating, the capacitance is usually found appreciably reduced [108], while the impedance at low frequencies (|Z|_0.01_ Hz) is significantly higher in comparison to the neat epoxy resin [73,125,135]. In particular, the higher the impedance, the better the corrosion resistance. Corrosion of coated substrates is influenced by absorption and diffusion of the corrosive agent into the coating, which mainly depends on the presence of voids or defects, but also on the adhesion of the coating to the metallic substrate [22,92,122,125]. The corrosion resistance is enhanced when the nanoparticles fill the micro-pores in the epoxy polymer, promoting a tortuous diffusion path, similar to a “labyrinth effect”, able to hinder the infiltration of corrosive agents into the coating [70,73,121,125,136].

The highest adhesion strength exhibited by the nanofilled epoxy coatings allows achieving excellent anti-corrosion performance [92,134,137]. Although low contents of nanoparticles can lead to small modifications in anti-corrosion properties, an excessive amount of nanofiller can promote aggregation of nanoparticles, with a consequent decrease in the corrosion resistance of the coating [108,121,123]. This behavior may be due to the formation of air pockets or voids in the matrix because of the presence of nanoparticles’ clusters in the interfacial layers [69]. Negative surface charges on the nanoparticles in the epoxy matrix, strongly reducing the diffusion of the negative ions (e.g., Cl^−^ and OH^−^), can further improve the barrier properties against corrosive agents [124,137].

The addition of functionalized nanoparticles has been found effective in reducing the tendency to aggregate, thus enhancing the properties of the nanocomposite, especially the corrosion resistance of epoxy coatings [21,92,93,120,121,124,137]. For example, graphene oxide functionalized with acrylate phosphorus functional monomer improved the corrosion protection of an epoxy coating, due to the increased thickness-to-diameter ratio of the nanoparticles which became more suitable to block the micropores in the neat epoxy resin [21]. On the other hand, more numerous connections with the epoxy matrix can occur with a surface modification of the nanoparticles, stronger interactions causing increases in strength and minimal defects in the interfacial areas [20,66,107].

The reduced barrier performance against corrosion can also shorten the nanocomposite’s service life [108]. In fact, mechanisms similar to those taking place in anti-corrosion processes also affect water absorption [110]. The effects of moist environments on the interfacial adhesion greatly depends on the nanofiller’s surface wettability [138] since hydrophobic features limit the water adsorption at the interface. Therefore, the use of hydrophobic nanofillers reduces the chemical interactions with water molecules and, in the absence of defects, lower amounts of water are absorbed and vapor barrier properties are enhanced [128]. As a consequence, the addition of nanofillers allows improvement of the stability and durability of the nanocomposite in humid environments [110,115].

Contact between the particles of nanofiller is necessary to form thermal conduction pathways; in this condition, the heat flow is promoted and the thermal conductivity increases [5,16,86,87]. The interfacial interaction is also an important factor since, where weak interactions are developed, the connection between the nanoparticles can be lost and thermal conduction is less efficient [45]. Significant conductivity values are reached when appropriate closeness between adjacent nanoparticles is achieved; for instance, the appropriate distance between CNTs has been found to be less than 2 nm [127].

**Figure 10:**
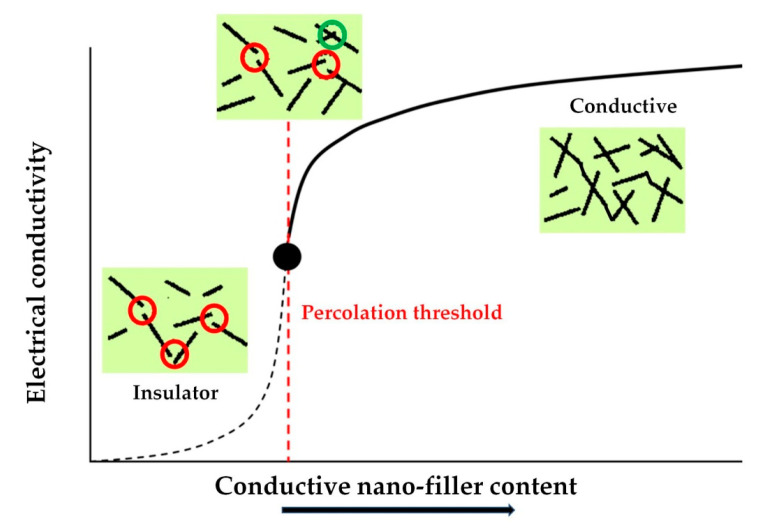
Evolution of electrical conductivity in nanocomposites.

Additionally, the high electrical conductivity in these nanocomposites is promoted by an effective direct electrical contact between the nanoparticles [15,110] (Figure 10). Such a condition can be more easily achieved for an intermediate degree of nanoparticles’ alignment rather than in randomly dispersed or perfectly parallel nanofillers [139]. Epoxy nanocomposites undergo an insulator-to-conductor transition when a conductive filler (among others, carbon nanotubes, expanded graphite, or graphene nanoplatelets) is added [110,127]. An electron tunneling mechanism between the nanoparticles mainly regulates the electrical conduction in the epoxy nanocomposites at a nano-scale level [127]. This effect positively affects the electrical properties since conduction takes place at lower loadings of nanofiller than those involving intimate contact between the nanoparticles [140,141], even if a suitable tunneling distance is necessary to provide conduction; for example, CNTs in polymer matrices have a maximum tunneling distance of 1.8 nm [142]. The presence of uniformly dispersed nanofillers is, once again, a basic requirement [101] and a critical loading, the so-called electrical percolation threshold (Figure 10), is needed to obtain electrical conductivity [9,15,17,109,110,127,139,143,144]. The percolation threshold is usually achieved using 0.5–1 wt.% loads of nanofiller, but nanoparticles’ functionalization can further decrease the required loading content [100,102].

The addition of nanofillers to epoxy resins enhance the thermal stability of the material. In most cases, higher final decomposition temperatures [93] and improved flame retardancy are observed. The nanocomposite becomes protected by a thicker surface layer of char residue which acting as oxygen barrier [79], retardant of mass and heat transfer [80], as well as hindering the release of pyrolysis products [16], is able to prevent further oxidative decomposition [93] or burning [43,75,79,81]. As already discussed, phosphorus-based nanofillers are highly efficient as flame retardants, but lower decomposition temperatures are observed when they are added to epoxy resins [81].

## 6. Applications of Nanofilled/Nanostructured Epoxy Resins

The extraordinary chemical and physical properties of a wide variety of experimental nano-epoxies enable a wide range of applications, varying from coatings to structural adhesives, from FRP matrix to electrical/electronic components. Epoxy nanocomposites have found advantageous application in many industrial fields [59,120] such as aerospace [114], aircraft [119], automotive [33,110], buildings [18,67,145,146], electronic devices [17,86,99,128], sensors [90,110,115,127,144] (even for biomedical applications), anti-static materials [9], abrasive tools [103], anti-corrosive coatings (for example in marine environment [147] or pipes [10]), and structural applications [29,68,109] with self-sensing properties [127]. Often, the improved properties allow an efficient usage for multifunctional purposes [27,69,148].

The nanofilled epoxy coatings display exceptional corrosion resistance performance and superior barrier properties due to the presence of the nanofiller that can limit the ingress of water and/or potentially harmful chemicals. The light weight and the high corrosion resistance of epoxy nanocomposites make these materials particularly advantageous in the aeronautical and automotive fields. The reinforcing effect of the nanofiller can also improve the superficial characteristics of the coating (wear and scratch resistance, hardness) with a substantial improvement in dirt resistance. When optical clarity and transparency are important features for the superficial treatment, a wide selection of nanoparticles, with a size smaller than the wavelength of visible light, is available in order to ensure these characteristics.

In the field of adhesives, the presence of well-dispersed nanoparticles in epoxy enhances the adhesion to different inorganic substrates, even when the cross-linking reactions of the resin are carried out in an uncontrolled environment (i.e., the resins are cold-cured). This represents a distinctive advantage in the field of adhesive resins employed in building industry where, for economical and practical reasons, the cure at high temperatures is not possible. 

The addition of carefully selected nanophases in an epoxy matrix for FRP is also capable to increase the bond at fiber/matrix interface, limiting debonding under applied stress, and the fracture toughness. Furthermore, the nanofiller can also supply different functional properties to fiber-reinforced composite for specific applications, along with greater resistance to environmental agents and fire. Once again, all these aspects are particularly desirable in the field of (building, aeronautical, and wind energy) construction.

Although the epoxy nanocomposites are more frequently employed because of their good electrical properties, they are also suggested as insulating materials [52,149]. Some nanofillers found an application for electrostatic discharge purposes (e.g., in aircraft applications). For instance, graphene and carbon nanofillers have been added to reduce static charge accumulation on polymer dielectric surfaces [9]. High thermal conductivity is another fundamental property of the nanofilled epoxies which can assure good durability and stability of electronic devices [5].

Nanostructured hybrid epoxies containing interpenetrating silica nanodomains represent a very promising solution to overcome limits and deficiencies displayed by typical epoxy-based adhesives and coatings, especially in terms of greater glass transition temperatures, superior resistance to weathering, and to harsh external conditions. Generally speaking, all nanofilled epoxy materials display improved durability than the pristine epoxy against water/moisture, temperatures, harsh environments, and fire (the addition of nanofillers can prevent flashover and the spreading of flame), allowing an extension of the lifetime of the applications in which the materials are employed.

## 7. Limits in the Production of Epoxy-Based Nanocomposites

As illustrated in the previous discussion, the effectiveness of an epoxy-based nanocomposite, realized with a functional nanomaterial, mainly depends on the chemical nature and the composition of the nanofiller and on the production/application methods. The latter, in particular, are established once adequate procedures and technologies have been identified to obtain optimal performance and characteristics of the nanocomposite. Consequently, greater production temperatures, longer processing times, use of toxic chemicals, and expensive techniques may be necessary, with processes having stricter purity requirements and lower yields: all these choices are feasible only for the production of high values components (for instance, in medicine). Furthermore, some methods/procedures are quite difficult to transfer from laboratory to industrial scale.

Different technological limits can be experienced in the production of polymer-based nanocomposites. The importance of the achievement of a fine and uniform dispersion of the nanofiller in the resin matrix has been widely discussed. The most widely used and economic method for the preparation of a nanocomposite is to simply disperse the nanofiller in the liquid resin, but this procedure is not always effective. In addition, when very low amounts of nanoparticles are added to the epoxy, it is still possible to employ the traditional methods for the production/application of the nanocomposite. On the other hand, since the viscosity of the nanofiller/resin suspension appreciably grows at high loadings of the nanoparticles, more sophisticated and expensive techniques must be used in the latter cases for the production of nanocomposites [83]. Once again, the production cost/performance ratio of such nanocomposites must be taken into account and put in relation to the economic value of the intended application.

Analogously, the experimental routes proposed for the functionalization of the nanofiller surface frequently request specially customized or finely synthesized chemicals or solvents while the adoption of cheap and commercially available chemical products would be more favored by industry, thus accelerating the scale-up manufacture.

Last, but not least, several concerns are associated with health issues and environmental impacts of such nanomaterials during their whole “life”, according to life cycle analysis (LCA) concepts. In the production of nanofillers, there is wide use of toxic compounds, acid/basic chemicals, and organic solvents. Furthermore, most of the formulations are proprietary of the suppliers, being not completely revealed their composition and potential hazards on humans, animals, and the environment [150]. The chemical reactions commonly employed to modify the surface of the nanofillers not only require high consumption of energy and chemicals but also produce severe pollution. In addition, due to their recent introduction to the market, if any, the knowledge of their potential long-term risks for human health, their interactions with the environment, and impacts on traditional waste cycle management are still limited. In conclusion, researchers and future producers should identify more environmentally friendly, but still efficient, approaches to take advantage of multifunctional nanocomposites limiting the occurrence of harmful effects for human health and the ecosystem.

## 8. Conclusions and Future Prospects

In this paper, an overview of the very recent papers (from 2016) that have appeared in the literature dealing with the main current developments and progress in the field of epoxy-based nanocomposites has been presented. The different nanofillers able to provide (alone or in combination with other nano-materials) improved properties to epoxies or to mitigate some of their well-known deficiencies have been introduced, highlighting the role played by the chemical nature of the nanoparticles, their size and aspect ratio, and their content in the epoxy. A different class of nanocomposite, i.e., the organic–inorganic hybrid systems, with the organic epoxy phase interconnected at the nano-scale level to the inorganic one, has been also illustrated. The reviewed papers have demonstrated that the development of an excellently performing nanocomposite based on epoxy resin, as well as on any other polymeric matrix, relies on the uniform dispersion of nanoparticles and on their interactions with the epoxy matrix; chemical modifications of the surface of the nanofillers allow achievement of these goals, but they are also able to alter the curing kinetics of the epoxy resin. The rate of advancement of cross-linking reactions, the final *T*_g_, and degree of cross-linking of the epoxy nanocomposite are also influenced by the kind of nanofiller, its content, and dimensional features. The huge potential of epoxy-based nanocomposites in many different applications has been described, providing some examples of the possible industrial uses of these nanomaterials. The main technological limits in the production of epoxy-based nanocomposites and their impact on human health and environment have been finally illustrated.

Smart epoxy nanocomposites have, then, proven to be invaluable materials for advanced applications in many fields; the exploitation of these nanomaterials in further new and demanding applications has probably not yet been revealed. However, research and development efforts should be still spent to produce safer materials, with new properties and excellent functional abilities. Thus, the production and application processes of such polymer-based nanomaterials must be carefully analyzed, identifying any route to optimize operational and safety segments.
